# Prognostic Role of Androgen Receptor in Triple Negative Breast Cancer: A Multi-Institutional Study

**DOI:** 10.3390/cancers11070995

**Published:** 2019-07-17

**Authors:** Shristi Bhattarai, Sergey Klimov, Karuna Mittal, Uma Krishnamurti, Xiaoxian (Bill) Li, Gabriela Oprea-Ilies, Ceyda Sonmez Wetherilt, Ansa Riaz, Mohammed A. Aleskandarany, Andrew R. Green, Ian O. Ellis, Guilherme Cantuaria, Meenakshi Gupta, Upender Manne, Johnson Agboola, Brett Baskovich, Emiel A. M. Janssen, Grace Callagy, Elaine M. Walsh, Anurag Mehta, Atika Dogra, Tanuja Shet, Pooja Gajaria, Tiffany Traina, Haruna A. Nggada, Abidemi Omonisi, Saad A. Ahmed, Emad A. Rakha, Padmashree Rida, Ritu Aneja

**Affiliations:** 1 Department of Biology, Georgia State University, Atlanta, GA 30303, USA; 2 Department of Pathology and Laboratory Medicine, Emory University, Atlanta, GA 30322, USA; 3 International Consortium for Advancing Research on Triple Negative Breast Cancer, Georgia State University, Atlanta, GA 30303, USA; 4 Department of Pathology, Emory University School of Medicine, Atlanta, GA 30322, USA; 5 Nottingham Breast Cancer Research Centre, Division of Cancer and Stem Cells, School of Medicine, University of Nottingham, Nottingham City Hospital, Nottingham NG5 1PB, UK; 6 Department of Gynecologic Oncology, Northside Hospital Cancer Institute, Atlanta, GA 30342, USA; 7 Department of Pathology, Piedmont Newnan Hospital, Newnan, GA 30265, USA; 8 Department of Translational Anatomic Physiology, University of Alabama at Birmingham, Birmingham, AL 3300, USA; 9 Morbid Anatomy and Histopathology Department, Olabisi Onabanjo University, Ogun 112106, Nigeria; 10 Department of Pathology, University of South Alabama College of Medicine, AL 36688, USA; 11 Department of Pathology, Stavanger University Hospital, 4002 Stavanger, Norway; 12 Discipline of Pathology, NUI Galway, Lambe Institute for Translational Research, Costello Road, H91 TK33 Galway, Ireland; 13 Research Department, Rajiv Gandhi Cancer Institute and Research Centre, Delhi 110-085, India; 14 Department of Pathology, Tata Memorial Hospital, Mumbai 400 012, India; 15 Department of Oncology, Memorial Sloan Kettering Cancer Center, New York, NY 90710, USA; 16 Department of Human Pathology, Faculty of Basic Clinical Sciences, College of Medical Sciences, University of Maiduguri, Maiduguri, Borno state 1069, Nigeria; 17 Department of Anatomic Pathology, Ekiti State University, Ado-Ekiti PMB 5363, Nigeria; 18 Department of Pathology, Ahmadu Bello University, Zaria, Kaduna State 810241, Nigeria

**Keywords:** androgen receptor, triple-negative breast cancer, prognosis, multi-institutional study

## Abstract

Background: The androgen receptor (AR) has emerged as a potential therapeutic target for AR-positive triple-negative breast cancer (TNBC). However, conflicting reports regarding AR’s prognostic role in TNBC are putting its usefulness in question. Some studies conclude that AR positivity indicates a good prognosis in TNBC, whereas others suggest the opposite, and some show that AR status has no significant bearing on the patients’ prognosis. Methods: We evaluated the prognostic value of AR in resected primary tumors from TNBC patients from six international cohorts {US (*n* = 420), UK (*n* = 239), Norway (*n* = 104), Ireland (*n* = 222), Nigeria (*n* = 180), and India (*n* = 242); total *n* = 1407}. All TNBC samples were stained with the same anti-AR antibody using the same immunohistochemistry protocol, and samples with ≥1% of AR-positive nuclei were deemed AR-positive TNBCs. Results: AR status shows population-specific patterns of association with patients’ overall survival after controlling for age, grade, population, and chemotherapy. We found AR-positive status to be a marker of good prognosis in US and Nigerian cohorts, a marker of poor prognosis in Norway, Ireland and Indian cohorts, and neutral in UK cohort. Conclusion: AR status, on its own, is not a reliable prognostic marker. More research to investigate molecular subtype composition among the different cohorts is warranted.

## 1. Introduction

Triple-negative breast cancer (TNBC) is an aggressive subtype characterized by the lack of estrogen (ER), progesterone (PR), and Her2 receptors. This designation masks the heterogeneity of this patient population and the challenge of stratifying them for optimal treatment selection [1,2]. Due to the paucity of treatment targets, cytotoxic chemotherapy is still the standard of care for TNBC, and there is a need to develop new and more effective targeted treatments for these patients. Among several therapeutic targets currently under study for the management of TNBC is the androgen receptor (AR) [3,4,5]. The AR, a nuclear steroid hormone receptor, is expressed in 10–43% of TNBCs [6,7]. In the absence of ERα, AR drives “luminal-like” gene expression patterns. One of the TNBC molecular subtypes consistently identified via gene expression profiling is the Luminal Androgen Receptor (LAR) subtype [3,4]. LAR TNBCs express full-length AR mRNA and AR target genes at high levels; however, they tend to be less proliferative and generally respond poorly to chemotherapy in both the neoadjuvant [8,9] and adjuvant settings [10]. Thus, AR expression reclassifies TNBCs into AR-positive TNBCs and AR-negative TNBCs. Because LAR TNBCs are dependent on AR signaling for their growth, AR-driven TNBC is considered an actionable subtype and targeting the AR pathway is an area of active investigation [11,12].

Studies have explored the prognostic role of AR in TNBC to better understand androgen action in TNBC, identify actionable factors that drive outcomes, and determine if testing for AR status should become part of routine clinical practice for TNBCs. However, there are conflicting reports about AR’s prognostic value in TNBC. While some studies report that AR-positivity is associated with better prognosis [13,14], others either contend that an AR-positive phenotype portends worse long-term outcomes [15,16] or that AR status has no significant impact on TNBC prognosis [17,18]. To some extent, these discrepant results can be attributed to small sample sizes, differences in the ethnic composition of cohorts, the anti-AR antibodies used for staining, staining/scoring method, and use of different thresholds to define AR-positivity. Together these factors make comparisons of results across studies and cohorts challenging. In this study, we evaluated the prognostic role of AR in 1,407 TNBC tumors from seven ethnically and racially diverse populations. The tumor samples were processed identically, and the differences in treatment protocols were accounted for.

## 2. Results and Discussion

**Figure 1:**
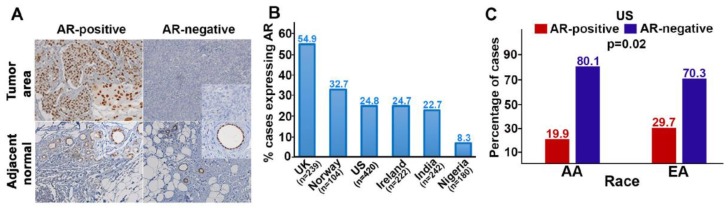
Androgen receptor (AR) expression in the seven cohorts of our multi-institutional study. (**A**) Representative micrographs showing AR expression in triple-negative breast cancer (TNBC) and their adjacent normal tissues, AR (brown) and nuclei (blue). Insets: 20× magnification. (**B**) Expression of AR in the different study cohorts. (**C**) Expression of AR among African Americans (AAs) and European Americans (EAs) in the US cohort. The *p*-value shows the significant difference in AR expression among AAs and EAs.

AR expression varied widely in our cohorts (from 8.3% in the Nigerian TNBCs, to 55% in the UK cohort). In the US cohort, 25% of all cases were AR-positive: Twenty percent among African Americans (AA) and thirty percent among those of European decent (EA; *p* = 0.02; Figure 1). This finding is consistent with previous reports [19]. The diversity in AR-positivity suggests our global cohorts could differ substantially in their TNBC molecular subtypes.

**Figure 2:**
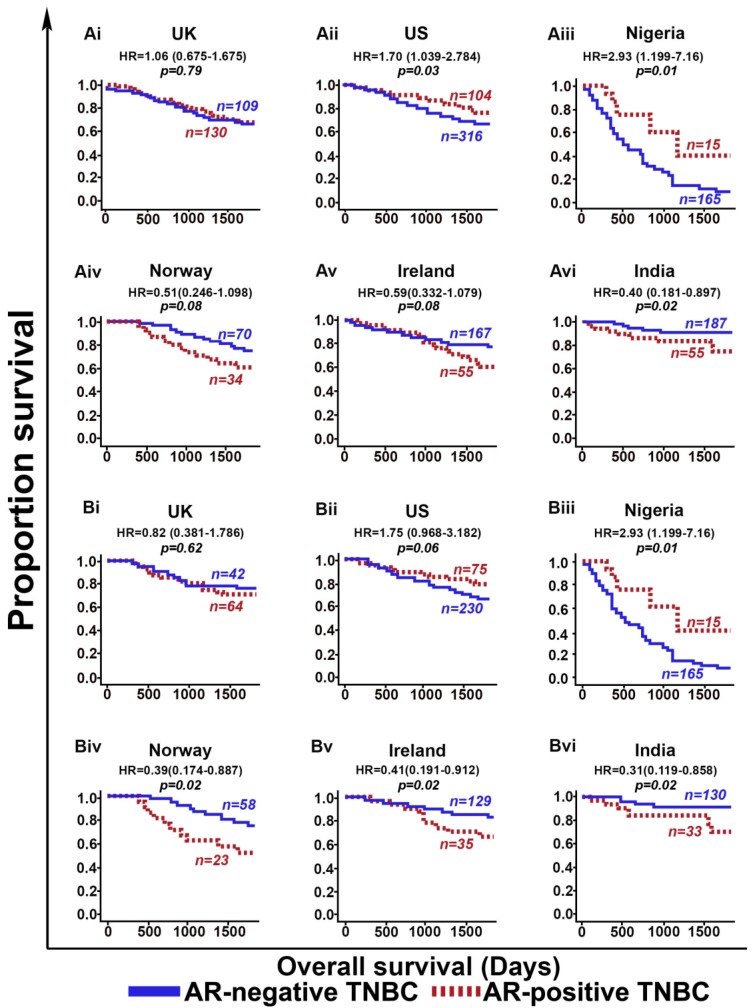
Prognostic significance of the androgen receptor (AR) in different study cohorts. (**A**) Kaplan Meier survival curves for AR-negative TNBC (blue) and AR-positive TNBC (red) patients from (i) UK overall cohort, (ii) US overall cohort, (iii) Nigeria overall cohort, (iv) Norway overall cohort, (v) Ireland overall cohort, and (vi) India overall cohort. (**B**) Kaplan Meier survival curves for adjuvant-chemotherapy-treated AR-negative TNBC (blue) and AR-positive TNBC (red) patients from (i) UK cohort, (ii) US cohort, (iii) Nigeria cohort, (iv) Norway cohort, (v) Ireland cohort, and (vi) India cohort.

Population-specific differences between cohorts became more apparent upon evaluating the prognostic value of AR among the cohorts in our study. We found that AR-positive TNBCs showed better OS than AR-negative TNBCs in the US (*p* = 0.03) and Nigerian (*p* = 0.01) cohorts among all patients (Figure 2A), as well as among adjuvant chemotherapy-treated patients (Figure 2B). By contrast, AR-positive TNBCs showed poorer prognosis (OS) than AR-negative TNBCs in the cohorts from Ireland (*p* = 0.08), Norway (*p* = 0.08), and India (*p* = 0.02) There was no statistically significant difference in OS between AR-positive TNBCs and AR-negative TNBCs in the UK (*p* = 0.79) cohort among all patients, or among adjuvant chemotherapy-treated patients (Figure 2A,B).

These survival trends did not change appreciably when the cut-point for AR-positivity was changed from 1% to 10%, or when an optimal cut-point was used to see if changing the cut-point affected the prognostic trend (Table S1). Multivariable Cox regression analyses that adjusted for potentially confounding variables, such as age, grade, chemotherapy, and population revealed two main trends. (1) In the US and Nigerian cohorts (wherein AR-positive TNBCs have a better prognosis compared with AR-negative TNBCs), the only variables significantly and independently associated with OS were being AA, being native African, and AR positive/negative status. (2) In the cohorts from Norway, Ireland, and India (wherein AR-positive TNBCs have a poorer prognosis compared with AR-negative TNBCs), age, being from India, and AR positive/negative status were all significantly and independently associated with OS. Thus, in five TNBC cohorts from four continents, AR positive/negative status shows population-specific patterns of association with OS (Table 1). One cohort showed no pattern of association with OS.

Some noteworthy limitations of our study include potential cohort-to-cohort differences in tissue fixation and the lack of centralized AR staining for some of the cohorts. Notwithstanding these caveats, we believe that by rigorously addressing previously reported inconsistent results regarding AR’s prognostic role in TNBC, our multi-institutional study has allowed the TNBC field to move forward.

We have demonstrated that AR’s prognostic value likely hinges on the proportions of the TNBC molecular subtypes (especially non-LAR subtypes) present in the cohorts, and perhaps other covert modifiers of AR biology. Candidate modifiers in different populations might include patients’ biogeographic ancestry, AR splice variants, epigenetic factors, tumor microenvironment, and repeat length polymorphisms (RLPs) in the AR gene. For example, among the alternatively spliced transcripts known to be generated from the AR gene, the constitutively active variant AR-V7 is often expressed in TNBC cells along with the full-length mRNA, but it regulates a transcriptional program distinct from full-length AR, and may affect outcomes. At present, little is known about the expression of AR-V7 and other splice variants in TNBC tumors in different populations. Few studies have examined the RLPs or tumor microenvironment in AR positive/negative TNBC tumors and correlated these features with the tumors’ molecular subtypes. Moreover, amplification of and mutations in the AR gene have been shown to drive castration- resistant prostate cancer; thus, further analysis of the mutational status of the AR gene and its downstream signaling components would shed some light on the biology of AR in different TNBCs arising in different populations. The aforementioned under-studied potential modifiers of AR biology could affect not only patients’ prognosis following chemotherapy, but also responses to AR-targeting agents, and are, therefore, very clinically relevant.

Another potential confounder of our results may be the molecular apocrine (MA) nature of the TNBCs in our cohorts. Studies have shown that AR binds ER-binding cis-regulatory elements in MA tumors and drives expression of genes normally regulated by ER. More importantly, a study of 58 transcriptionally defined MA tumors found that a significant proportion of these tumors showed expression of AR mRNA via qRT-PCR even though they were AR-negative via IHC [20]. These intriguing findings raise the possibility that some of the TNBC tumors in our cohorts (as well as in cohorts used in previous studies) that were classified as AR-negative based on IHC, may, in fact, be MA tumors that express detectable levels of AR mRNA and show activation of AR-target genes. In other words, IHC-based detection of AR expression may not be sensitive enough to identify all TNBC tumors in which AR-mediated signaling is active, nor to identify patients likely to respond to the AR-targeted treatments. Moreover, studies have shown AR as an independent predictor for the complete pathological response in breast cancer [21]. Since all our study cohorts are comprised of the adjuvant chemotherapy-treated patients and patients who did not receive any systemic therapy, it will be worth exploring the prognostic and predictive role of AR in similarly diverse cohorts in the context of neoadjuvant treatment.

## 3. Materials and Methods

### 3.1. Study Cohorts and Samples

All aspects of this study were approved by Institutional Review Boards of the institutions involved (IRB number: H19306). Materials and data were shared in accordance with the stipulations of Material Transfer Agreements and Data User Agreements between Georgia State University (GSU) and the other participating institutions as listed in Table S2). A total of 1407 cases consecutively diagnosed with TNBC were identified from the electronic health records of multiple institutions (Table S2). Samples were collected from institutions in the US (*n* = 420), UK (*n* = 239), Norway (*n* = 104), Ireland (*n* = 222), Nigeria (*n* = 180), and India (*n* = 242). Resection samples from primary tumors were formalin-fixed and paraffin-embedded (FFPE). Samples from Nigeria, US, and Norway were processed at GSU, and the samples from the UK, India and Ireland were processed at their respective institutions.

Clinicopathological data (Table 2; reviewed and provided by a pathologist from the respective hospitals), race information (for US cohort only), overall survival (OS), and deidentified tissue blocks were available for all cases. All patients in the Nigerian cohort received adjuvant chemotherapy; by contrast, the remaining cohorts included a mix of patients who did not receive any systemic treatment in the adjuvant setting and patients who received adjuvant chemotherapy. Patient consent was not required, because all samples were archival.

### 3.2. Immunohistochemistry (IHC)

All samples in the six cohorts were stained using the following protocol: Briefly, the FFPE TNBC primary tumor resection samples were deparaffinized following by rehydration in a series of ethanol baths (100%, 90%, 75%, and 50%). Heat-induced retrieval of antigen epitopes was performed in citrate buffer (pH 6.0) using pressure cooker at 15 psi for 30 min. Next, the samples were quenched using hydrogen peroxide for 20 min, followed by blocking with the Ultra-Vision protein block (Life Sciences, Fremont, CA, USA) for 10 min. Samples were then incubated for 60 min at room temperature with anti-AR primary antibody (Monoclonal Mouse Anti-Human Androgen Receptor, clone AR 441, DAKO) at 1:50 dilution. Next, the samples were incubated with MACH2 HRP-conjugated secondary antibody (BioCare Medical, Pacheco, CA, USA) for 30 min. The AR antigen was visualized using the Betazoid 3, 3-diaminobenzidine Chromogen Kit (BioCare Medical). The tissue sections were counter stained with Mayer’s hematoxylin for 1 min. Slides were then dehydrated in alcohol, cleared in xylene, and mounted with mounting medium. Appropriate negative and positive controls were used during staining.

### 3.3. Assessment of IHC Staining

Nuclear AR staining is indicative of active receptors, because AR can translocate to the nucleus upon ligand binding. Therefore, the stained slides were scored for the percentage of tumor-cell nuclei that showed AR-positivity. TNBC samples were considered AR-negative if they had <1% of the tumor nuclei positive for AR; samples that had AR in ≥1% of the nuclei were considered AR-positive. Samples from the Nigerian, US, and Norwegian cohorts were stained and scored centrally (at GSU) and the samples in the UK, Indian, and Irish, cohorts were stained and scored at the respective institutions by two independent pathologists without prior knowledge of the patients’ pathologic or outcome data. For cross validation of the IHC scores, stained slides of grade- and stage-matched cases (representing 20–30% of each cohort) were selected from each cohort (along with the positive and negative control slides from each round of staining). The slides were reviewed and re-scored at Nottingham University Hospital by two pathologists who were blinded to the clinical annotation and previous IHC scores. The scores from the second round of scoring showed >99% concordance with the original IHC scores.

### 3.4. Statistical Analyses

Bar graphs were plotted using Excel and SPSS. Experimental groups were compared using Student’s t-test, and p-values were calculated with *p* < 0.05 considered statistically significant. Statistical analyses were carried out with SAS 9.4^®^ software (Cary, NC, USA). Differences between clinicopathological proportions were determined using the χ^2^ test. Differences between baseline IHC biomarker expressions or continuous clinicopathological variables were evaluated via a 2-tailed *t*-test. Univariate Kaplan-Meier curves were used to investigate the effect of AR status (positive vs. negative) on OS, and the log-rank test was used to assess the statistical significance of between-group survival differences. Multivariable Cox Proportional Hazard models were used to adjust for grade, chemotherapy, population, and age with significance being determined with the Wald chi-square test. For all clinical survival analysis, we used the OS, which was calculated as the time interval from surgery until death, and death was used as an event.

## 4. Conclusions

Our findings, therefore, provide a strong impetus to pursue future studies that integrate transcriptomic and genomic information to better parse and refine the classification of TNBCs. Deeper dissection of the pathways upstream/downstream of AR, as well as transcriptomic, genomic, and epigenetic profiling of samples from different patient populations, could move us from the currently used coarse categories towards finer signatures. Signatures that may reclassify AR positive/negative TNBCs into more clinically actionable subgroups.

## Figures and Tables

**Table 1:** Multivariate analysis to reveal population-specific differences between cohorts. AA = African American; AR = androgen receptor. Bold refers to significant *p*-values.

Multivariate Cox Regression Analysis of Common Clinicopathological Variables and AR					
Variables		US and Nigeria Study Cohorts		Norway, Ireland and India Study Cohorts	
		Hazard Ratio (95% CI)	*p*-value	Hazard Ratio (95% CI)	*p*-value
Overall Survival					
Age	<50 vs. ≥50	1.00 (0.99–1.01)	0.3934	1.03 (1.01–1.05)	**<0.001**
Grade	2	0.77 (0.86–8.84)	0.085	0.25 (0.05–1.17)	0.0801
Grade	3	3.63 (1.15–11.44)	0.0275	0.37 (0.09–1.56)	0.1777
Adjuvant Chemotherapy	treated vs. non-treated	0.72 (0.39–1.32)	0.2877	0.91 (0.56–1.47)	0.7026
Population	AA	1.82 (1.30–2.53)	**0.0004**	-	-
Population	African	11.22 (8.11–15.51)	**<0.0001**	-	-
Population	Indian	-	-	0.49 (0.27–0.89)	**0.0201**
Population	Irish	-	-	0.79 (0.48–1.28)	0.3462
AR	AR-positive vs. AR-negative	1.70 (1.10–2.61)	**0.0157**	0.58 (0.37–0.90)	**0.0154**

**Table 2:** Patient demographic and tumor data stratified by cohort; *n* (%).

Clinicopathological Variables of Study Cohorts						
Variables	UK	Norway	Ireland	US	Nigeria	India
	***n* = 239**	***n* = 104**	***n* = 222**	***n* = 420**	***n* = 180**	***n* = 242**
Age						
<50	128 (53.55)	45 (43.3)	55 (24.8)	129 (30.7)	97 (53.9)	122 (50.4)
≥50	111 (46.45)	59 (56.7)	167 (75.2)	291 (69.3)	83 (46.1)	120 (49.6)
Clinical stage						
I/II	212 (88.7)	91 (87.5)	177 (79.7)	320 (76.2)	NA	NA
III/IV	26 (10.9)	11 (10.6)	34 (15.3)	84 (20)	NA	NA
Missing	1 (0.4)	2 (1.9)	11 (5.0)	16 (3.8)	NA	NA
Grade						
1	5 (2.1)	1 (1.0)	2 (0.9)	11 (2.6)	7 (3.9)	0 (0)
2	12 (5.0)	15 (14.4)	31 (14)	79 (18.8)	68 (37.8)	13 (5.4)
3	221 (92.5)	83 (79.8)	189 (85.1)	322 (76.7)	101 (56.1)	228 (94.2)
Missing	1 (0.4)	5 (4.8)	0 (0)	8 (1.9)	4 (2.2)	1 (0.4)
Chemotherapy						
Treated	106 (44.4)	81 (77.9)	164 (73.9)	305 (72.6)	180 (100)	163 (67.4)
Untreated	110 (46)	20 (19.2)	58 (26.1)	56 (13.3)	0 (0)	46 (19.2)
Missing	23 (9.6)	3 (2.9)	0 (0)	59 (14.1)	0 (0)	33 (13.4)
Vital status						
Dead	112 (46.9)	44 (42.3)	56 (25.2)	131 (31.2)	139 (77.2)	28 (11.6)
Alive	127 (53.1)	60 (57.7)	166 (74.8)	289 (68.8)	41 (22.8)	214 (88.4)
Androgen Receptor						
Positive	130 (54.9)	34 (32.7)	55 (24.77)	104 (24.8)	15 (8.3)	55 (22.7)
Negative	109 (45.1)	70 (67.3)	167 (75.3)	316 (75.2)	165 (91.7)	187 (77.3)

## Supplementary Materials

The following are available online at https://www.mdpi.com/2072-6694/11/7/995/s1, Table S1: Table showing overall survival of AR positive TNBC at different thresholds of AR, Table S2: Table representing number of cases and sample type evaluated for AR expression in global TNBC cohort and diverse institutions from US.

## Abbreviations

AR = androgen receptor; TNBC = triple-negative breast cancer; US = United States of America; UK = United Kingdom; ER = estrogen receptor; PR = progesterone receptor; LAR = luminal androgen receptor; OS = overall survival; FFPE = formalin-fixed and paraffin-embedded; GSU = Georgia State University; IHC = immunohistochemistry; AA = African American; EA = European American; RLP = repeat length polymorphisms; MA = molecular apocrine.
