# Supplementary material for: Prognostic Role of Androgen Receptor in Triple Negative Breast Cancer: A Multi-Institutional Study
Source: Cancers (Basel). 2019 Jul 17;11(7):995. doi: 10.3390/cancers11070995 (PMC6678933; doi:10.3390/cancers11070995)
Supplement: Supplementary file 1 [file cancers-11-00995-s001.pdf]

Supplementary Materials: Prognostic Role of Androgen Receptor in Triple Negative Breast Cancer: A Multi-Institutional Study

Shristi Bhattarai, Sergey Klimov, Karuna Mittal, Uma Krishnamurti, Xiaoxian (Bill) Li, Gabriela Oprea-Ilies, Ceyda Sonmez Wetherilt, Ansa Riaz, Mohammed A. Aleskandarany, Andrew R. Green, Ian O. Ellis, Guilherme Cantuaria, Meenakshi Gupta, Upender Manne, Johnson Agboola, Brett Baskovich, Emiel A. M. Janssen, Grace Callagy, Elaine M. Walsh, Anurag Mehta, Atika Dogra, Tanuja Shet, Pooja Gajaria, Tiffany Traina, Haruna A. Nggada, Abidemi Omonisi, Saad A. Ahmed, Emad A. Rakha, Padmashree Rida and Ritu Aneja

Table S1. Table showing overall survival of AR positive TNBC at different thresholds of AR.

Different Threshold Analysis for AR-Positive TNBCs										
	5% Cutoff			10% Cutoff			Optimal Cutoff			
Study	AR+ TNBC	AR- TNBC	*p*-value	AR+ TNBC	AR- TNBC	*p*-value	AR+ TNBC	AR- TNBC	*p*-value	Optimal
Cohorts	Survival	Survival		Survival	Survival		Survival	Survival		Cutoff (%)
US	82.42	70.21	0.0402	82.5	70.59	0.0471	82.98	69.94	0.0248	2
UK	70.27	67.19	0.3711	70.53	67.36	0.3677	73.33	66.46	0.1613	30
Nigeria	85.71	22.54	0.0846	80	23.43	0.2682	68.75	20.73	0.0117	2
Ireland	60.34	75.43	0.0738	60.78	74.9	0.1127	58.93	75.64	0.0472	6
Norway	63.33	77.03	0.0533	60.71	77.63	0.0211	57.69	78.21	0.0064	2
India	75	91.55	0.0157	75	91.24	0.0304	80.77	92.06	0.0212	1

Table S2. Table representing number of cases and sample type evaluated for AR expression in global TNBC cohort and diverse institutions from US.

Description of Study Cohorts and Samples			
Study Cohorts	No. of Cases	Sample Type	Years of Diagnosis
**UK**	**239**	TMA	1987–1998
Nottingham Breast Cancer Research Center			
**Norway**	**104**	Full-Face	1978–2004
Stavanger University Hospital			
**Ireland**	**222**	TMA	2000–2015
NUI Galway, Lambe Institute for Translational Research			
**US**	**420**		2000–2014
Emory University Hospital	142	Full-Face	
Grady Memorial Hospital	114	Full-Face	
Northside Hospital Cancer Institute	95	Full-Face	
West Georgia Medical Center	32	Full-Face	
University of Alabama at Birmingham	23	Full-Face	
University of South Alabama College of Medicine	14	Full-Face	
**Nigeria**	**180**		2000–2008
Olabisi Onabanjo University	77	Full-Face	
University of Maiduguri	16	Full-Face	
Ekiti State University	45	Full-Face	
Ahmadu Bello University	42	Full-Face	
**India**	**242**		2005–2014
Tata Memorial Hospital	139	TMA	
Rajiv Gandhi Cancer Institute and Research Center	103	Full-Face	

© 2019 by the authors. Licensee MDPI, Basel, Switzerland. This article is an open access article distributed under the terms and conditions of the Creative Commons Attribution (CC BY) license (http://creativecommons.org/licenses/by/4.0/).
